# Therapeutic Innovations: Tyrosine Kinase Inhibitors in Cancer

**DOI:** 10.3390/vetsci3010004

**Published:** 2016-01-20

**Authors:** Nikolaos Dervisis, Shawna Klahn

**Affiliations:** Virginia Maryland College of Veterinary Medicine, 245 Duck Pond Dr., Blacksburg, VA 24061, USA; klahn@vt.edu

**Keywords:** tyrosine kinase inhibitors, comparative, cancer, chemotherapy, dog, cat, human

## Abstract

Conventional cytotoxic chemotherapy involving DNA-interacting agents and indiscriminate cell death is no longer the future of cancer management. While chemotherapy is not likely to completely disappear from the armamentarium; the use of targeted therapies in combination with conventional treatment is becoming the standard of care in human medicine. Tyrosine kinases are pivotal points of functional cellular pathways and have been implicated in malignancy, inflammatory, and immune-mediated diseases. Pharmaceutical interventions targeting aberrant tyrosine kinase signaling has exploded and is the second most important area of drug development. The “Valley of Death” between drug discovery and approval threatens to blunt the enormous strides in cancer management seen thus far. Kinase inhibitors, as targeted small molecules, hold promise in the treatment and diagnosis of cancer. However, there are still many unanswered questions regarding the use of kinase inhibitors in the interpretation and management of cancer. Comparative oncology has the potential to address restrictions and limitations in the advancement in kinase inhibitor therapy.

## 1. Introduction

Kinases are among the first oncogenes identified. Initial understanding of oncogene function originates from experiments utilizing retroviruses expressing oncogenic kinases. Murine, feline, and avian retroviruses were the first to demonstrate integration into the host genome, thereby transducing genes harbored at the insertion site. Src kinase is transduced within the avian genome by the Rous sarcoma virus and is the driving force behind Rous sarcoma. Similarly, Raf kinase is transduced by the same retrovirus. Activation of genes encoding kinases contribute to oncogenic transformation of the infected cell. An array of over 500 kinases have since been identified using animal tissue culture models and genetic methodologies [1,2]. Their role in tumorigenesis, tumor progression, and metastasis has been expanding, along with pharmacological attempts to therapeutically intervene the aberrant functions.

## 2. Digital Age in Cellular Biology

Kinases catalyze the transfer of the gamma-phosphate group of ATP onto a substrate. Kinases mediate nearly all signal transductions, thereby regulating multiple cellular activities such as proliferation, survival, apoptosis, metabolism, transcription, differentiation [3,4]. Accumulating pharmacological and pathological evidence demonstrate that kinases are promising drug targets for the treatment of numerous diseases, such as cancer, inflammatory diseases, central nervous system disorders, cardiovascular diseases, and diabetes [5]. The catalytic activity depends on the phosphorylation status of specific amino-acid residues. Conformational and other changes induced by phosphorylation events result in dramatic shifts in the kinase catalytic activity, manifesting as a binary switch in functional behavior of the enzyme [6,7,8].

Kinases are located at key positions within the cell. Intercellular communication and the extracellular environment are monitored through signal receptors, while intracellular communication is conducted through secondary signaling molecules and at specific functional checkpoints. Individual subcircuits representing relative simplified functions, such as cellular growth and energy metabolism, have multiple interconnections and crosstalk, forming a functional signaling network that is characterized by higher-level complexity [9]. Kinases have an important role in the amplification and termination of signals, facilitating communication between integral parts of the cell, and between cells. Thus, kinases can be thought as the molecular receptors, amplifiers and transducers of extra and intracellular signals, facilitating the cell’s response to the challenges of life.

A consequence of this complex and intricate signaling network is that kinases exhibit pleiotropy and locus heterogeneity. Mutations within the same gene may result in several phenotypes, while the same disease phenotype can sometimes result from mutations in different kinase genes. For example, gain-of-function mutations in the region of the immunoglobulin domain of fibroblast growth factor receptor 1 (FGFR1) result in Pfeiffer syndrome and osteoglophonic dysplasia, whereas loss-of-function mutations throughout the protein cause hypogonadotrophic hypogonadism [10,11].

## 3. Disease as a Kinase Dysfunction

Despite the enormous amount of research and rapid developments during the past decades, cancer continues to be a worldwide killer. In the USA, cancer accounts for 23% of the total deaths, and is the second most common cause of death after heart disease. It is expected to surpass heart disease as the leading cause of death in the next few years [12,13]. The intense research in the molecular biology of cancer resulted in an explosion of candidate genes that drive oncogenesis. Nearly simultaneous with the discovery of cancer-associated genes, strategies to repair them were explored. Early attempts focusing on gene replacement therapies were not translated into clinical use due to technological, ethical, and biological barriers. Efforts were soon turned towards pharmacologic methods of blocking specific aberrant oncogenic signaling.

Mutations in protein kinases are overrepresented approximately four-fold compared with a random selection of genes, and reportedly the most frequently mutated family of genes contributing to neoplastic diseases [14,15]. Protein kinases may act as tumor suppressors or proto-oncogenes in normal, healthy cells. Aberrations in protein kinase function can lead to tumorigenesis through numerous mechanisms, including the activation of proliferative pathways, genomic instability, reduction of the DNA damage response, deactivation of apoptotic pathways and/or the promotion of angiogenesis and cellular motility [15]. Gain-of-function mutations typically increase constitutive kinase activity, leading to unrestrained cellular signaling, while loss of-function mutations can lead to a loss in cell signaling. The combined signal aberrations can have a profound effect on the cell, leading to oncogenesis, tumor growth and metastasis.

## 4. Kinases as an Attractive Drug Target

Protein kinases are considered to be the second most important group of drug targets after G-protein-coupled receptors [16,17]. Aberrant kinase activity is implicated in a large variety of diseases, particularly those involving inflammatory or proliferative responses, such as cancer, rheumatoid arthritis, cardiovascular and neurological disorders, asthma and psoriasis. Directly or indirectly, hundreds of human diseases have been connected to protein kinase dysfunction. The ability to modulate kinase activity therefore represents an attractive therapeutic strategy for the treatment of human illnesses [18].

Kinase activity is typically regulated by interconverting between two structural conformations via phosphorylation of key amino acid residues, shifting the balance between active and inactive. These two states are characterized by movements in conformationally mobile loops that border or block the ATP binding site of the kinase. For this reason, the dissociation constant for ATP may be significantly higher for the inactive conformation than for the active conformation. This kinase activation model provides the framework for drugs designed to interact with specific kinase domains. A large number of kinase inhibitors selectively target the inactive conformation, whereas other compounds bind to both conformations with similar affinity [19,20,21]. Inhibitors that bind to the inactive conformation likely face weaker competition with cellular ATP. The potential result is enhanced activity *in vivo*, acting primarily to shift equilibria between conformational states to prevent kinase activation, indirectly inhibiting activity [22].

**Figure 1:**
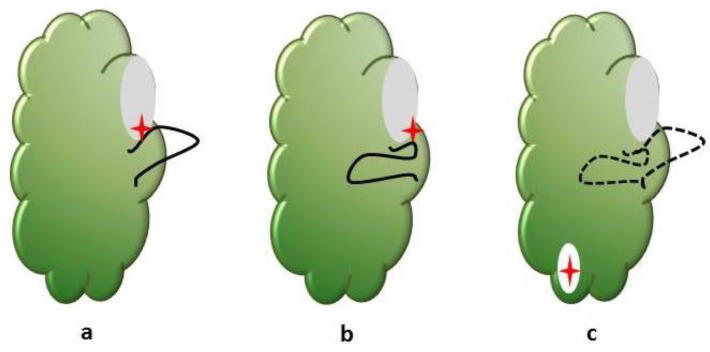
The activation loop of the protein kinase domain regulates access to the ATP binding site. The conformation of a conserved Asp-Phe-Gly (DFG) motif within the activation loop is used to categorize the binding mode of inhibitors. The common types of kinase—kinase inhibitor interactions include: (**a**) Type I inhibitors (**red** star) bind the ATP binding site (grey) of the protein kinase domain (green). The aspartate side chain in the conserved DFG motif at the beginning of the activation loop (black) faces into the active site; (**b**) Type II inhibitors bind a flipped conformation of the DFG motif in which the aspartate side chain faces outwards; (**c**) Allosteric ligands bind to binding pockets (white) that do not overlap with the active site of the kinase. The DFG motif conformation is not important. These binding pockets can be adjacent to the active site or distant from the active site.

The modulation of kinase activity can be achieved through either direct or indirect strategies (Figure 1). Imatinib (Gleevec; Novartis) is the prototype of direct protein-tyrosine kinase inhibitors that inhibits the BCR-ABL phosphorylation activity through blocking ATP binding. Imatinib has been approved for the treatment of patients with BCR-ABL positive chronic myeloid leukemia (CML) and patients with Kit (CD117)-positive gastrointestinal stromal tumors. BCR-ABL is the constitutively active tyrosine kinase in CML and in certain forms of acute lymphoblastic leukemia. Imatinib also inhibits the kinase activity of platelet derived growth factor receptor, stem-cell factor receptor and c-kit. Indirect kinase inhibition involves disruption of protein-protein interactions. Cetuximab (Erbitux; ImClone/Bristol-Myers Squibb) is a monoclonal antibody that selectively binds to the extracellular domain of human epidermal growth factor receptor (EGFR), and competitively inhibits binding of epidermal growth factor (EGF) to its receptor tyrosine kinase. Bevacizumab (Avastin; Genentech) is the first anti-angiogenesis cancer drug approved by the FDA. Bevacizumab binds to human vascular endothelial growth factor A (VEGF-A), and prevents it from binding to its receptor tyrosine kinases, Flt-1/VEGFR1 and KDR/VEGRF2. Inhibition of VEGF signaling interferes with tumor blood vessel development, a process that is crucial for tumor growth and metastasis [23].

Compounds that bind to protein kinases outside of the ATP-binding pocket may possess advantages over ATP-competitive counterparts. Drugs can be administered at concentrations closer to their biochemical inhibition constant, as inhibition is not affected by fluctuations in the cellular concentration of ATP. There is a potential for greater selectivity as residues outside the ATP binding pocket tend to be less conserved. In certain cases, noncompetitive inhibitors can be substrate selective, inhibiting the activity of a kinase against only a subset of its targets. Rapamycin, the first noncompetitive kinase inhibitor to be identified, is a cyclic macrolide that inhibits the protein kinase mammalian target of rapamycin (mTOR) [24]. MEK1 inhibitor PD098059, the first synthetic noncompetitive kinase inhibitor to be described, acts by binding to inactive MEK1 and preventing its phosphorylation by the upstream kinase Raf [25,26]. Several such inhibitors, termed allosteric, have been described, targeting kinases such as Akt, and inhibitor of nuclear factor kappa-B kinase (IKK-2) [27,28].

The target regions for most kinase inhibitors are intracellular. As small molecules, kinase inhibitors enter the cell by diffusion down the concentration gradient that exists across the membrane. The rate at which this process occurs, and the rate of active efflux, are important determinantsof the intracellular bioavailability of a kinase. How quickly the intracellular inhibitor concentration reaches a steady state and the relative concentrations of inhibitor inside and outside the cell at steady state rely on the relative lipophilicity *vs.* hydrophilicity characteristics of the drug. The rate that it permeates the cellular membrane and non-specific binding to proteins and may affect the route of administration and dosing schedule [29,30]. Expression of drug efflux pump transporters has been shown to reduce the steady-state intracellular drug concentration, although most mammalian cells in culture appear to have less efflux activity for kinase inhibitors compared to chemotherapeutics such as doxorubicin.

## 5. Hitting the Target

The transition from cytotoxic chemotherapy to molecularly targeted cancer drug discovery and development has resulted in an increasing number of successful therapies that have impacted the lives of a large number of cancer patients. The BCR-ABL inhibitor imatinib is generally regarded as a trailblazer drug that most impressively validated the concept of designing a small molecule therapeutic. Imatinib is designed to treat a defined patient population with chronic myeloid leukemia in which the malignancy is driven by the BCR-ABL translocation. The improvement in survival has been dramatic [31]. These successes were followed by a number of other small molecule drugs inhibiting critical cancer targets, essentially changing the anti-neoplastic armamentarium of modern Oncology. There has been an explosion of approved kinase inhibitors, with nearly 20 drugs approved by the FDA in a four-year period (Table 1).

Veterinary Oncology has been slowly following the fast pace of human Oncology, and there are currently three kinase inhibitors approved by FDA, and a number of small molecular inhibitors under various stages of development (Table 2).

The clinical success of such targeted therapeutics depends to a large degree to the availability of predictive markers. The specific histopathologic diagnosis may not be of such importance, as the presence of markers associated with specific genetic aberrations (mutations, internal tandem duplications, gene amplifications) [32,33,34,35]. For example, the anaplastic lymphoma kinase (ALK) was first implicated in the pathogenesis of an aggressive type of non-Hodgkin’s lymphoma [36]. Rearrangements of the ALK gene were subsequently discovered to be are present in 3% to 5% of non-small-cell lung cancers (NSCLC) [37]. These genetic lesions define a distinct subgroup of NSCLC that typically occurs in younger patients who have never smoked or have a history of light smoking [38]. Knowing the presence of these rearrangements can guide the treatment protocol selection, alter the prognosis, and essentially result in a truly personalized approach in cancer therapy [29,35].

**Table 1:** Approved tyrosine kinase inhibitors in human medicine, 2011–2015 ^a^.

Drug	Target	Year Approved	Indication
Vandetanib (Caprelsa^®^)	Flt1, Flt4, KDR, EGFR, Ret	2011	MTC
Crizotinib (Xalkori^®^)	ALK, MET, EML4-ALK fusion protein	2011	NSCLC
Ruxolitinib (Jakafi^®^/Jakavi^®^)	JAK1, JAK2	2011	Myelofibrosis
Vemurafenib (Zelboraf^®^)	BRAF	2011	Melanoma
Bosutinib (Bosulif^®^)	BCR/ABL1	2012	CML
Axitinib (Inlyta^®^)	Flt1, Flt4, KDR, Kit, PDGF-Rα/β	2012	RCC
Cabozantinib (Cometriq^®^)	KDR, Mek	2012	MTC
Regorafinib (Stivarga^®^)	KDR, TEK	2012	CC, GIST
Ponatinib (Iclusig^®^)	BCR/ABL1	2012	CML, ALL
Dabrafenib (Tafinlar^®^)	BRAF	2013	Melanoma
Trametinib (Mekinist^®^)	MEK1, MEK2	2013	Melanoma
Afatinib (Gilotrif^®^)	ERBB2, EGFR	2013	NSCLC
Ibrutinib (Imbruvica^®^)	BTK	2013	MCL, CLL
Tofacitinib (Xeljanz^®^)	JAK3	2013	Rheumatoid arthritis
Idelalisib (Zydelig^®^)	PI3-K	2014	CLL
			Follicular B-cell NHL
Ceritinib (Zykadia^®^)	ALK	2014	ALK^+^ NSCLC
Lenvatinib (Lenvima^®^)	VEGFR2 and VEGFR3	2015	Radioactive iodine-refractory DTC
Palbociclib (Ibrance^®^)	CDK4 and CDK6	2015	Breast carcinoma

^a^ Abbreviations: ALL, acute lymphoblastic leukemia; CC, colorectal cancer; CML, chronic myelogenous leukemia; DTC, differentiated thyroid cancer; GIST, gastrointestinal stromal tumor; MCL, mantle cell lymphoma; MTC, medullary thyroid carcinoma; NHL, Non-Hodgkin’s lymphoma; NSCLC, non-small-cell lung carcinoma; RCC, renal cell carcinoma.

**Table 2:** Approved tyrosine kinase inhibitors in veterinary medicine, all drugs.

Drug	Targets	Year Approved	Indication
Toceranib (Palladia^®^)	VEGF-R2	2009	Patnaik grade 2 or 3, recurrent, cutaneous mast cell tumors with or without regional lymph node involvement in dogs
	PDGF-Rα		
	Kit		
	Flt-3		
	*RET*		
	*JAK family*		
Masitinib (Kinavet-CA1^®^)	Kit	2010 ^a^	Nonresectable grade 2 and 3 cutaneous mast cell tumors in dogs that have not previously received radiotherapy and/or chemotherapy except corticosteroids
	PDGF-Rα/β		
	Lyn		
	FGF-R3		
Oclacitinib (Apoquel^®^)	JAK1	2013	Control of pruritus associated with allergic dermatitis and control of atopic dermatitis in dogs at least 12 mos of age
	JAK 2		

^a^ Conditional approval by the FDA expired in December 2015 and the drug is not commercially available in the US. The drug can only be obtained through the FDA’s personal import mechanism.

## 6. Missing the Point

The high specificity of the kinase inhibitors appears to be their Achilles heel. As many of the kinase inhibitors exert their tumoricidal effects primarily by inhibiting a specific kinase, there is a strong selective pressure for cells to acquire resistance through mutations in the kinase gene that abrogate drug binding. Additional non-mutation kinase inhibitor resistance mechanisms have been documented, including target amplification and upregulation of alternative kinase pathways such as hepatocyte growth factor receptor in the acquisition of resistance to EGFR kinase inhibitors in lung cancer [30,39,40]. The rapid proliferation of cancer cells and the acquisition of mutations conferring drug resistance has become a recurring theme in the clinic. To date the most extensive clinical and laboratory characterization of resistance-causing mutations has been performed for BCR–ABL in the context of imatinib and second-generation inhibitors. Furthermore, it has been shown in specific hematological tumors that quiescent stem cells are refractory to tyrosine kinase inhibitors, and these cell populations may be instrumentally involved in resistance mechanisms [41].

Inhibitor resistance conferred by mutations at the gatekeeper residue appears to be the common theme for a variety of kinases. The gatekeeper residue term refers to the amino acid side chain at the position that determines the relative accessibility of the hydrophobic pocket located near the ATP/drug binding site. Access to this pocket is important to many kinase inhibitors because hydrophobic interactions in this site are crucial for the binding affinity of the drug. Several strategies are being investigated to overcome kinase inhibitor resistance mutants. A first approach was to develop inhibitors that can tolerate diverse amino acids at the gatekeeper position [42,43]. A second approach is to target the kinase with inhibitors that bind at alternative binding sites [44]. A third approach involves targeting other molecules that may be required for signal transduction in the specific kinase pathway [45]. These approaches have been demonstrated to work in cell culture, and rodent models and clinical trials are currently underway.

## 7. Unanswered Questions

Considerable progress made with the new molecularly targeted therapies. Significant advances have occurred in the treatment of diseases in which few treatment options are available, such NSCLC and melanoma. However, for many patients the therapeutic options are still limited, and the process of bringing a new drug to patients is frustratingly slow with very high failure rates [46,47]. The point of failure between basic research and new drug approval has often referred to as the “Valley of Death” [48,49].

The current model of targeted drug development is severely flawed. The inefficiency of translating seemingly important discoveries from the lab to the clinic can be demonstrated by a simple comparison between the research publications on kinase function and the drug approvals. Multiple obstacles within the current framework severely restrict the timely exploration of combinatorial kinase inhibitor therapies. Regulatory, patent secrets, and risk-averse corporate cultures need to be quickly remedied in order to be prepared, as cancer is becoming the leading cause of death in both humans and veterinary patients.

Scientific questions potentially affecting patient clinical management and treatment options include:

*I*  When to treat with kinase inhibitors?
*II*  What is the biologically sound way to combine kinase inhibitors with cytotoxic chemotherapy?
*III*  How to combine multiple kinase inhibitors in a treatment protocol
*IV*  How to determine efficacy when tumor size is not an appropriate measure
*V*  How to manage treatment failure? Switch to different kinase inhibitors, or add different kinase inhibitors to the current therapy?

The canine cancer patient can help in answering some of these questions. Spontaneous cancers in pet dogs offer a unique and largely unexploited translational research opportunity for cancer imaging, device and drug development. Dogs suffer from spontaneous malignancies at similar rates and types as humans. Limitations with rodent models stem from development from inbred strains of mice kept in controlled environments. Companion animals, like humans, are genetically diverse and are exposed to many of the same environmental influences. Furthermore, purebred dogs represent unique gene pools, with genetic predisposition to cancers segregating within specific breeds. This provides the opportunity to study complex phenotypes in a relatively simplified genetic background, allowing the identification of critical genetic events that drive malignant behavior.

Dogs suffering from cancer have been successfully used in elegantly designed studies, providing useful and translatable pharmacokinetic/pharmacodynamic data [50,51,52,53,54,55]. Data from canine clinical trials have informed on the scheduling and dosing of small molecular inhibitors in human cancer patients [56]. The availability of the canine genome and growth of high throughput genomic technologies and informatics has enabled comparative oncology to describe canine cancer biology and define potential therapeutic targets in many of the same ways as human cancers [57,58]. Canine cancer patients offer an unparalleled resource for targeting the tumor stroma and tumor/host immune interactions, due to the presence of syngeneic host-tumor relationship and a competent immune function [50].

Using spontaneous pet dog oncology modeling does not require up-front treatment with specific cancer treatment regimens. Thus, novel therapeutic agents can be offered through clinical trials at any stage in cancer presentation. The disease progression times in pet dogs is compressed, allowing for evaluation of a variety of pharmacodynamics intervention outcomes and providing longitudinal endpoints of cancer response through serial sampling. Randomized controlled trials can be conducted in the newly-diagnosed, adjuvant, or metastatic settings, evaluating the utility of drug combinations and drug selection algorithms across a range of tumor and genetic background scenarios [51].

Finally, the infrastructure and resources to realize the potential of the canine cancer patient as an additional tool in conquering cancer exists. The National Cancer Institute’s Comparative Oncology Trials Consortium (NCI-COTC; http://ccr.cancer.gov/resources/cop/COTC.asp) brings together study sponsors with academic veterinary oncology centers in North America to support multicenter clinical trials of investigational therapeutics [59]. High-quality biologic samples (tumor, normal, biologic fluids) from dogs with various malignancies are available via the Pfizer-Canine Comparative Oncology and Genomics Consortium (CCOGC) biospecimen repository (www.ccogc.net) [60]. As a direct result of such mechanisms drugable target identification and signaling pathway similarities have been identified between canine and human cancer patients [61,62,63,64,65].

## 8. Future Directions

The use of pet dogs in translational medicine may be the “bridge” to the Death Valley [66]. Translational medicine is the process of applying knowledge gained from basic biomedical research to clinical practice. It involves bridging new research findings, scientific discoveries and new techniques to approaches in the prevention, diagnosis or treatment of diseases. The ultimate outcome is the patient’s health. The paradigm shift is that scientific discoveries should not only be celebrated in basic laboratories and research institutes, or published (and archived) in prestigious medical journals—they need to make their way to hospitals, clinics, and into patients’ lives to be real successes. Translational medicine involves a longer-term strategy for research that is beyond the immediate outcomes of a research project [67].

A major hurdle in therapeutic development is the translation of efficacy determined during the drug discovery phase into efficacy in the clinic. There are often discrepancies between drug efficacy, demonstrated in the current preclinical experimental models, and final efficacy, or lack thereof, in patients. Tissue culture and various rodent models have been and are used as potential predictive models for response in human cancers. It remains to be decided which model is most predictive, however, it is clear that none can reliably predict significant clinical efficacy.

## 9. Concluding Remarks

Veterinary cancer patients can provide the solution for some of the current limitations faced in targeted therapies. Several of the features that define human cancer, including long periods of latency, the complex biology of cancer recurrence and metastasis, the stromal interactions, and outcomes to novel therapies are frequently not adequately represented in murine cancer [68]. Dogs develop cancer and are diagnosed, staged, and treated in an almost identical manner to human cancer patients. Canine tumors develop in a complex genetic background, in the backdrop of intact immune system, and are exposed to the same environmental carcinogens as their human counterparts. Many dogs are essentially family members. Their owners are motivated to treat their pets, and as advocates for their pet, they actively seek participation in Clinical Trials. With the continuous improvement of the canine genomic annotation, dogs with cancer may represent an excellent opportunity for bridging the Valley of Death. One of the payoffs for human cancer patients is reduced exposure to ineffective clinical trials. The partnership under One Medicine of basic science researchers, veterinary and human oncologists, and pharmaceutical industry, has significant potential to advance drug discovery and approval. Ultimately, this ideal could result in improved cancer management, regardless of species.
